# A New Medical Therapy for Multiple Endocrine Neoplasia Type 1?

**DOI:** 10.17925/EE.2022.18.2.86

**Published:** 2022-08-23

**Authors:** Hessa Boharoon, Ashley Grossman

**Affiliations:** Neuroendocrine Tumour Unit, ENETS Centre of Excellence, Royal Free Hospital, London, UK

**Keywords:** Dihydroorotate dehydrogenase, leflunomide, multiple endocrine neoplasia type 1, neuroendocrine tumour, pancreas, therapy

## Abstract

Pancreatic neuroendocrine tumours (pNETs) are a major manifestation of multiple endocrine neoplasia type 1 (MEN1), and the most significant cause of morbidity and mortality in this disorder. There is some evidence that the early use of somatostatin analogues can retard progression, especially of small non-functioning tumours, but there are no other prophylactic therapies for patients, and the treatment of metastatic disease is similar to that for sporadic pNETs. A recent study has shown that in cell line and animal models, *MEN1* mutations lead to an upregulation of the enzyme dihydroorotate dehydrogenase (DHODH), which is involved in increasing precursor metabolites for the synthesis of pyrimidines. In these studies, blockade of this pathway by various means, including the DHODH inhibitor leflunomide, attenuates cell growth and tumour progression, suggesting a critical dependence on DHODH specifically in *MEN1*-mutated tissue. Preliminary clinical studies in three patients with MEN1 and pNETs have indicated some therapeutic potential of this drug, which has previously been used for some years in patients with rheumatoid arthritis. It is suggested that further clinical trials of this re-purposed drug are indicated to evaluate its potential for the treatment of patients with MEN1 and pNETS. This article describes the clinical problem of MEN1 and pNETs, and reviews the recent publication reporting on these initial results.

Multiple endocrine neoplasia type 1 (MEN1) is an inherited tumour endocrine syndrome, with the parathyroid glands, anterior pituitary gland and pancreas as the main sites of MEN1-related neuroendocrine tumours (NETs). MEN1 is inherited in an autosomal dominant manner, with 90% of individuals diagnosed with this disease having an affected parent, while only 10% have a *de novo MEN1* germline mutation.^1^ The protein product of *MEN1*, menin, is implicated in the regulation of transcription, genome stability, cell division and cell proliferation, although the exact role of menin in tumorigenesis has yet to be fully elucidated.^2^

In clinical terms, the parathyroid glands are most often affected in relation to clinical disease, while duodenal gastrin-secreting tumours are common and most often treated medically in the first instance.^1^ However, it is pancreatic NETs (pNETs), which are often multifocal, that are the major cause of morbidity and indeed mortality, as a substantial minority undergo malignant transformation with metastatic spread. In such cases, treatment is generally based on the standard protocols for other pNETs, which would include surgery, somatostatin receptor ligands, peptide receptor radionuclide therapy and chemotherapy, as well as molecular targeted agents such as sunitinib and everolimus.^3^ However, the only curative treatment is surgical, which is often problematic even at early-stage disease due to the multifocality of the tumours and hence the likelihood of recurrence.^4^ Furthermore, extensive surgery to avoid possible metastatic disease requires a total pancreatectomy, with all of its associated short- and long-term morbidity.^4^ Currently, pNETs are the leading cause of MEN1-related death.^1^

The high prevalence and malignant potential of pNETs in MEN1 emphasize the need for an evidence-based screening programme at an early stage to enable meticulous follow-up and timely intervention to prevent metastatic disease. Indeed, recent studies on large data collections have highlighted a detection rate of 70%–80% of pNETs in MEN1 using highly sensitive imaging modalities such as endoscopy, contrast-enhanced triphasic computed tomography scanning and magnetic resonance imaging.^5^

The MEN1 syndrome is caused by germ-line heterozygote inactivating mutations of the *MEN1* gene, which encodes menin, a tumour suppressor protein. MEN1-related pNETs develop following the complete loss of function of wild-type menin due to loss of heterozygosity, according to the Knudson two-hit hypothesis. Menin is a key regulator of endocrine cell plasticity, and its loss in these cells is sufficient for tumour initiation. Familial genetic screening of mutation-positive non-index patients reveals a high number of individuals who present relatively small lesions within the pancreas or who may even be negative at imaging at the time of an *MEN1* mutation diagnosis. These patients are then generally subject to specific routine MEN1-associated tumour follow-up protocols, using regular biochemical markers and imaging procedures.

**Figure 1:**
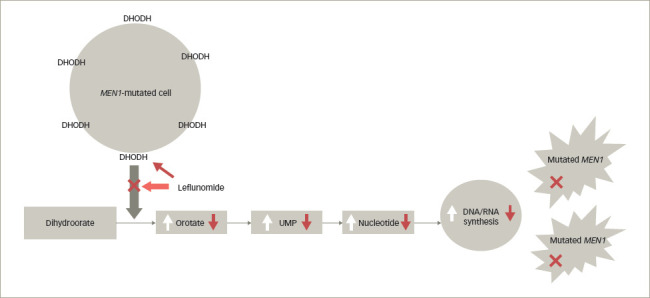
The effect of mutated *MEN1* on the expression of dihydroorotate dehydrogenase and the production of nucleotides, and the effect of leflunomide

*Mutated* MEN1 *increases the expression of DHODH. The white arrows show the changes seen in* MEN1*-mutated cells, the red arrows the effect of leflunomide.*

*DHODH = dihydroorotate dehydrogenase; MEN1 = multiple endocrine neoplasia type 1; UMP = uridine monophosphate.*

There are currently several recommendations for the management of patients with MEN1 with small non-functioning PNETs (NF-pNET). The National Comprehensive Cancer Center in the USA suggests a relatively conservative approach for tumours 1–2 cm in diameter (www.nccn.org, version 2.2014), whereas the French Endocrine Tumor Study Group also suggests a conservative management of NF-pNETs <2 cm provided there are no signs of aggressiveness such as rapid progression on imaging studies.^6^

When NF-pNETs achieve a size ≥2 cm or a growth rate >0.5 cm per year, pancreatic surgery is generally recommended. Patients with MEN1 with pNETs <2 cm in size are not recommended for surgery or medical therapy unless a functioning endocrine syndrome is present. However, despite these recommendations, metastatic spread has also been observed in patients with MEN1 with apparently indolent pNETs <2 cm.^1,3,4^

In terms of medical therapy that could be initiated at an early stage to avoid metastatic spread, somatostatin analogues represent the first therapeutic option in patients affected with low-grade (G1-G2) NETs. According to the current guidelines, somatostatin analogues should be considered in this setting for functioning endocrine syndromes not controlled by symptomatic therapies, and for metastatic unresectable pNETs or those with residual disease following partial resection. Nevertheless, in a *MEN1*-knockout (KO) mouse model, the somatostatin analogue lanreotide significantly delayed pNET progression.^7^ In addition, a retrospective clinical study reported that long-acting repeatable octreotide appeared to retard pNET progression in a series of 40 MEN1-associated pNETs <2 cm, with an objective response rate of 10%.^8^ Furthermore, in a prospective series of 42 patients from the same group, lanreotide autogel resulted in a significant increase in progression-free survival at a median follow-up of 73 months.^9^ Nevertheless, there has remained a distinct lack of other specific medical therapies for pNETs in patients with MEN1, either as prophylaxis against metastasis or to retard initial progression.

In this context, a very recent publication has raised the prospect of a defined medical treatment specifically targeted at patients with MEN1, which may significantly influence the progression of pNETs. Basic science studies have indicated a specific dependence of *MEN1*-mutated cells on an enzyme involved in pyrimidine synthesis, and positive results were seen in a limited number of patients treated with a re-purposed agent that blocks this enzyme.

## Paper review

A team led by Y. Jiao,^10^ working mainly from the Peking University Medical Center in Beijing, China, initially used clustered regularly interspaced palindromic repeats (CRISPR-Cas9) technology to perform synthetic lethal screening in the cell line U251. Of 19,114 genes screened, 26 were found to cause lethality when *MEN1* was knocked out, of which two, the trifunctional enzyme CAD (carbamoyl phosphate synthetase, aspartate transcarbamylase, dihydroorotase) and dihydroorotate dehydrogenase (DHODH), were especially potent. More specifically, *MEN1*-KO cell lines were found to over-express DHODH compared with either the wild-type cells or several other cell lines, leading to a depletion of dihydroorotate and a shift to orotate and orotate-related downstream products. These downstream products are important in the synthesis of pyrimidine nucleotides. These cells were more dependent on this pathway than wild-type cells, as small interfering RNA treatment of the *MEN1*-KO cells led to significantly more marked cell death compared with the wild type, essentially demonstrating ‘addiction’ to DHODH in *MEN1*-mutated cells.

DHODH is a mitochondrial enzyme that increases throughput to precursors for pyrimidine synthesis, vital in the production of nucleotides (*Figure 1*). This pathway is especially important in cells undergoing proliferation, but also in tumour cells undergoing therapeutic damage to DNA and requiring a new supply of nucleotides to restore such chemotherapeutic- or radiotherapeutic-induced damage. Germline mutations in the gene for DHODH lead to the rare Miller syndrome, which is associated with craniofacial and limb defects similar to those seen in children with foetal exposure to methotrexate.^11^ The overactivation of DHODH in *MEN1*-mutant cells appears to indicate particular dependence on this enzyme in such cells, and offers a relatively specific route to such oncogenic ‘addiction’. Indeed, a similar detrimental effect was demonstrated when these cells were treated with leflunomide,^12^ an inhibitor of DHODH (as well as certain other kinases), which is in clinical use in the treatment of rheumatoid arthritis and has also been suggested to have antineoplastic properties. Furthermore, the decrease in DHODH was shown to lead to loss of DHODH transcription and translation, probably due to changes in promoter accessibility in *MEN1*-KO cells.^10^ Importantly, the effect could be shown to be overcome by the addition of orotate, indicating that DHODH inhibition was probably its major mechanism of efficacy. *MEN1*-KO xenograft growth in nude mice was also attenuated by leflunomide, while tissue from an *MEN1*-KO mouse implanted into wild-type mice showed 20/20 with tumours in controls compared with 6/20 in leflunomide-treated mice. In an *MEN1* haploid KO (Men1+/-) mouse model, a model similar to patients with the MEN1 syndrome, 65% of mice with *MEN1* germline mutations developed naturally occurring tumours at 20 months of age; in the intervention group taking leflunomide from 6 months of age, the incidence was reduced to 5%.^10^ The results of the study demonstrate that leflunomide may be an effective drug for the treatment of *MEN1*-mutated tumours. Leflunomide also attenuated the spontaneous incidence of pNETS in the *MEN1*-KO mice, especially when treated early as opposed to its late addition.^10^

The article also reported that the first three patients with MEN1 enrolled in the study showed signs of improvement: one 43-year-old man had a progressive pNET that had been stable for 11 months; a second 39-year-old man had a recurrent pNET in the head of the pancreas, which ‘almost disappeared’ and showed diminished uptake on ^68^Ga-dotatate scanning; a third 40-year-old man demonstrating progressive disease on a somatostatin analogue and everolimus, showed stable disease for 8 months on leflunomide. No adverse events of leflunomide were reported.

Leflunomide has been used for the treatment of rheumatoid arthritis, with reputedly few adverse effects, and has suggested anti-tumour activity. The current preliminary results on patients with advanced pNETs demonstrated some clinical activity, although the animal studies indicate that the earlier in disease course that leflunomide is used, the more likely it is to be effective. From that point of view, it may be that this re-purposed agent, which is reputedly inexpensive, could play a more important role in the prophylaxis of pNETs in MEN1. The researchers suggested that this may be because the *MEN1* mutation is involved in tumorigenesis at an early stage in the process of tumour development. Leflunomide can achieve targeted killing of early tumours with a small size and few mutations, thus suggesting early therapeutic intervention and effective prevention. However, it might also be the case that used in conjunction with chemotherapeutic agents and poly-adenosine diphosphate-ribose polymerase inhibitors, which antagonize DNA repair, the decreased supply of nucleotides could offer synergistic therapeutic effects.^13^

## Conclusions

Summarizing the above results, this study may provide new ideas and strategies for the treatment of patients with *MEN1*-mutated tumours and the prevention of MEN1-induced lethality, which are important aims in current research.^1^ Nevertheless, these studies need to be repeated in other laboratories, and any clinical studies need to be carried out in larger groups of patients, with vigorous controls, possibly using a comparator such as a somatostatin analogue.
